# THE ROLE OF TRUST FOR DEMOGRAPHIC VARIABILITY IN VACCINE HESITANCY AND BEHAVIORS AMONG OLDER ADULTS

**DOI:** 10.1093/geroni/igad104.2846

**Published:** 2023-12-21

**Authors:** Andrea Huseth-Zosel, Heather Fuller, Melisa Hajdar, Emily Kinkade, Paul Carson

**Affiliations:** North Dakota State University, Fargo, North Dakota, United States; North Dakota State University, Fargo, North Dakota, United States; North Dakota State University, Fargo, North Dakota, United States; North Dakota State University, Fargo, North Dakota, United States; North Dakota State University, Fargo, North Dakota, United States

## Abstract

Because of increased risks of infectious disease, understanding older adults’ decisions related to trust and reception of vaccines is important. This study examines the associations between demographic characteristics, vaccine hesitancy, compliance, and the potential mediating role of uncertainty of who to trust. Seven hundred and thirty-nine older adults in North Dakota, oversampled from rural and lower vaccinated regions, completed a mailed survey about vaccine beliefs and behaviors. Participants reported demographic characteristics (age, gender, years of education, rurality, political ideology, and self-perceived physical health) and answered a 7-item scale of vaccine hesitancy. Vaccination behaviors were measured with an assessment of overall vaccine compliance, as well as measures of uptake for influenza, shingles, pneumonia, and SARS-CoV-2 immunizations. Uncertainty who to trust was assessed with the single-item “I am not sure who to trust when it comes to information about vaccines”, which was rated from Strongly Agree (4) to Strongly Disagree (1) and used as a mediator. Bootstrapping analyses indicated uncertainty of who to trust partially or fully mediated associations of rurality, education level, and political leaning with vaccine hesitancy. Further, trust partially or fully mediated associations of education level, health, and political leaning with vaccine behaviors; however, trust did not account for the association between vaccine hesitancy and behaviors. Findings highlight demographic variations in older adults’ vaccine beliefs and behaviors that may be partially explained due to uncertainty who to trust, indicating that practitioners should implement education and trust-building interventions, especially with rural-dwelling, conservative-leaning, and lower-educated older adults.

